# Simulated microgravity, Mars gravity, and 2g hypergravity affect cell cycle regulation, ribosome biogenesis, and epigenetics in Arabidopsis cell cultures

**DOI:** 10.1038/s41598-018-24942-7

**Published:** 2018-04-23

**Authors:** Khaled Y. Kamal, Raúl Herranz, Jack J. W. A. van Loon, F. Javier Medina

**Affiliations:** 1 Agronomy Department, Faculty of Agriculture, Zagazig University, Zagazig, Egypt; 2 Centro de Investigaciones Biológicas (CSIC), Ramiro de Maeztu 9, 28040 Madrid, Spain; 3 DESC (Dutch Experiment Support Center), Dept. Oral and Maxillofacial Surgery/Oral Pathology, VU University Medical Center & Academic Centre for Dentistry Amsterdam (ACTA), Gustav Mahlerlaan 3004, 1081 LA Amsterdam, The Netherlands; 4 ESA-ESTEC, TEC-MMG, Keplerlaan 1, NL-2200 AG Noordwijk, The Netherlands

## Abstract

Gravity is the only component of Earth environment that remained constant throughout the entire process of biological evolution. However, it is still unclear how gravity affects plant growth and development. In this study, an *in vitro* cell culture of *Arabidopsis thaliana* was exposed to different altered gravity conditions, namely simulated reduced gravity (simulated microgravity, simulated Mars gravity) and hypergravity (2g), to study changes in cell proliferation, cell growth, and epigenetics. The effects after 3, 14, and 24-hours of exposure were evaluated. The most relevant alterations were found in the 24-hour treatment, being more significant for simulated reduced gravity than hypergravity. Cell proliferation and growth were uncoupled under simulated reduced gravity, similarly, as found in meristematic cells from seedlings grown in real or simulated microgravity. The distribution of cell cycle phases was changed, as well as the levels and gene transcription of the tested cell cycle regulators. Ribosome biogenesis was decreased, according to levels and gene transcription of nucleolar proteins and the number of inactive nucleoli. Furthermore, we found alterations in the epigenetic modifications of chromatin. These results show that altered gravity effects include a serious disturbance of cell proliferation and growth, which are cellular functions essential for normal plant development.

## Introduction

Plants on Earth are subjected to a constant mechanical stimulation from the gravitational field, which has played a major role in their evolution. Gravity is the only parameter which has remained constant on Earth since life appeared on the surface of our planet, regarding both the direction and magnitude of the gravity vector^1,2^. All living organisms are well adapted to this 1 *g* level, which is used by plants to define their developmental pattern and to optimize the capture of light, water, and mineral salts. Thus, any changes of this parameters would cause significant physiological alterations, which would activate the adaptive response pathways. Understanding these changes is important for increasing our basic knowledge on plant physiology, and it is also essential for plant space biology if we consider that the magnitude of gravity is one of the most important factors differentiating the Earth environment from other celestial bodies. The same qualities that make plants essential to life on Earth, namely absorption of CO_2_, release of O_2_ and water vapor, and their use as a food source make them highly desirable on long-term human space missions, as essential components of “Bioregenerative Life Support Systems”. For this purpose, plants need to be adapted to grow in near-zero gravity (space) and fractional gravity, e.g., on the Moon, where the gravitational acceleration is 1/6 *g*, or on Mars at 3/8 *g*.

Spaceflights to the International Space Station (ISS) provide unique conditions to investigate plant biology in microgravity. However, research in the Near-Earth Orbit and access to ISS is severely constrained by the limited number of flight opportunities. For that reason, ground-based facilities (GBFs) for simulated microgravity are valuable tools for preparing spaceflight experiments and also for facilitating stand-alone studies. They provide an additional cost-efficient platform for gravitational research^3^. Several ground-based facilities have been developed, capable of providing a simulation of altered gravity environments^4^. For instance, a simulated reduced gravity environment can be simulated by 3D rotation of the biological object in a Random Positioning Machine (RPM, Airbus Defense and Space, Leiden, The Netherlands)^5,6^ and a Large Diameter Centrifuge (LDC) can indeed provide a stable hypergravity environment^7^.

Different types of plant biological systems, mostly from the model species *Arabidopsis thaliana*, have been used in microgravity investigations including plant seedlings^8–10^ and semisolid cell cultures^11–13^. Plant *in vitro* suspension cell culture is a powerful tool as a model in plant cell cycle studies of actively proliferating cells, a subpopulation that it is represented by some dozens of cells in the meristems of the plant^14^. Therefore, the choice of cell cultures allows the use of experimental and analytical approaches that require thousands of cells. Dispersed plant cell suspension cultures also allow the study of cell division in the absence of any further developmental process, by providing a homogenous population of near-identical cells^15^. Among the few available *Arabidopsis* cell cultures, a suspension culture of the fast-growing cell line MM2d was selected and maintained^16,17^ to be used in our study.

Since gravity is a driving force for plant development, the study of cell growth and proliferation mechanisms under real and simulated microgravity has general relevance, other than the specific interest of these research topics in solving current problems of space exploration and space biology. Indeed, the activation of modulators of cell growth and cell proliferation in meristems plays a key role in the regulation of plant development. Cell growth and cell proliferation are tightly interconnected to one another in actively proliferating cells, and the coordinated response of meristematic cell functions to developmental signals was called “meristematic competence”^18^.

In general, cell division is modulated through the regulation of cell cycle progression, which occurs at known checkpoints, and determines the rate at which cells divide^19,20^. The cell cycle is one of the most comprehensively studied biological processes, particularly given its importance for growth and development; indeed, the role of the cell cycle machinery during development remains an important scientific challenge^19^. A typical proliferating eukaryotic cell divides on average every 24 hours^21,22^. In turn, cell growth, in meristematic cells, expressly represents the production of cell biomass, mainly proteins, above a certain threshold compatible with cell division, which is subjected to a particular checkpoint^18,23^. In actively proliferating (cycling) cells, which, in plants, are meristematic cells, the concept of “cell growth” is different from “cell expansion”, and the difference is not semantic, but physiological. It has been demonstrated that not all processes involving an increase of the size of the cell should strictly be called “cell growth”, but this term should be reserved to those processes involving macromolecular (protein) synthesis. This process requires ribosome biogenesis and hence RNA pol I activity. This has been repeatedly demonstrated in a variety of proliferating cell systems, both plant and animal, and, specifically, in plant meristematic cells. A great deal of work has been devoted to provide experimental support to these arguments^18,23–27^. Consequently, meristematic cell growth is determined largely by the activity of ribosome biogenesis^24,25^. This occurs in a distinct nuclear domain, the nucleolus, whose structural features are a reliable marker of the rate of ribosome production^28^.

On the ISS, *Arabidopsis* root meristematic cells exposed to the microgravity environment resulted in the uncoupling of cell growth and cell proliferation^10^. Subsequent experiments performed on Earth with seedlings, using different facilities for simulation of altered gravity conditions, have shown similar trends as in spaceflight. Simulated microgravity affects the regulation of cell cycle progression, as shown by changes in the transcription of cyclin B1. The same experiments indicated a depletion of the nucleolar activity and ribosome biogenesis^8,10,29^. Alternatively, exposing *Arabidopsis* seedlings to hypergravity (2g), had less impact on plants than microgravity, as shown by the effects on cell growth and proliferation^8,30^. In other experiments on meristematic root cells grown in simulated microgravity, a depletion of ribosome biogenesis was reported^31,32^, but these findings were not connected to other cell functions. However, alterations in cell cycle regulation induced by real microgravity have been observed in other biological model systems, including human cell cultures^33^.

Furthermore, genomic and proteomic experiments with callus cell cultures of *Arabidopsis thaliana* in real and simulated microgravity, including microarray experiments, have also demonstrated alterations in the expression of genes and proteins involved in various processes, functions and cellular activities, some of them related to abiotic stress responses and cell cycle regulation^3,13,34^.

In addition to the effects induced by the microgravity environment, it would be interesting to explore the cellular response of proliferating cells to partial gravity (less than the nominal 1 *g* that occurs on Earth). This interest is related to space biology, and space exploration since these levels of partial gravity are found at the Moon (0.17 *g*) and Mars (0.37 *g*)^35^. Such studies also have a general physiological relevance since this investigation would allow the determination of thresholds for the biological response to gravitational alterations. In terms of our current levels of knowledge, the regulation of the cell cycle under partial gravity is understudied despite its future relevance.

Understanding the effects of an environmental change in reprogramming cellular functions and gene expression patterns should consider, necessarily, epigenetic mechanisms. Term of Epigenetics is used for the study of heritable changes in gene expression and activity that occur without any alteration of DNA sequence^36^. Much of today’s epigenetic research is converging on the study of covalent and noncovalent changes in DNA and histone proteins and the mechanisms by which such changes influence overall chromatin structure. Two major changes, responsible for epigenetic mechanisms, have been identified: methylation of the cytosine residues of DNA (DNA methylation) and different types of modification of histone proteins associated with DNA (histone modifications), the most important of which is acetylation^36–38^. Functionally, epigenetic mechanisms influence gene activity and expression through changes in the structural state of chromatin, which may have either positive or negative impact on gene transcription^39^. Chromatin structure is recognized as a highly dynamic and major player in cell cycle regulation, not only owing to the changes that occur as a consequence of cell cycle progression but also because some specific chromatin modifications are crucial to move across the cell cycle^40^. Moreover, recent studies reported that core cell cycle regulators control gene expression through histone modifications^41^.

Therefore, the purpose of this work is, firstly, to further validate *in vitro* plant cell culture as a model system to study alterations induced by gravity changes. Secondly, we aim to get a better understanding of the mechanisms operating at the level of individual cells in their response to the gravitational stress affecting cell growth and cell proliferation. We also consider the mechanisms altering the regulation of the cell cycle and the response to gravitational alterations through chromatin remodeling. Furthermore, we aim to extend our investigations to a wide range of gravitational levels, including two levels of simulated reduced gravity (simulated microgravity (µ*g*) and simulated partial gravity) and hypergravity (2g). In the case of partial gravity, simulated Mars gravity (0.37 *g*) will be used, with the purpose of achieving both basic knowledge about partial gravity effects as well as contributing to make possible a manned Mars exploration program.

## Results

### Gravitational alterations induced changes in the distribution of *Arabidopsis* cell cycle phases

**Figure 1:**
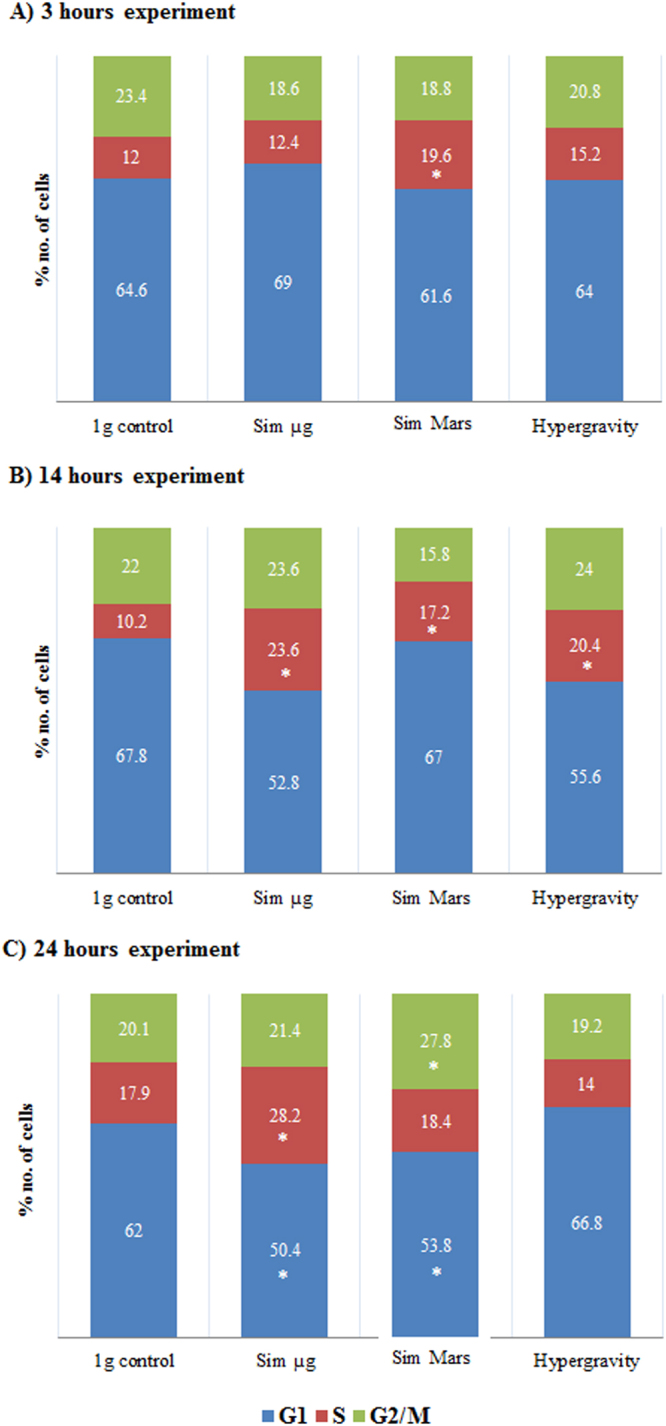
Distribution of cell cycle phases under simulated microgravity (Sim µ*g*), simulated Mars gravity, and hypergravity (2g), compared to 1 *g* control, for different times of exposure of the cell culture to each gravitational condition: (**A**) 3 hour. (**B**) 14 hour. (**C**) 24 hour. The relative percentages of the three cell cycle phases were obtained from graphics of DNA content after flow cytometry analysis of cells labeled with DAPI. The proportion of cells in G1 and G2/M phases were quantified from the respective G1 and G2/M peaks of the flow cytometry graphic diagrams, whereas the S-phase percentage was estimated as the remaining proportion of cells up to 100%. 10000 cells in three replicates were counted by flow cytometry. Significant differences versus 1 g control are shown (*). P-value > 0.05. Data including mean ± SE values for the experimental error estimated using three different biological replicates is available in the supplementary Table 3.

We have investigated the changes in different parameters of the cell cycle caused by the exposure of *Arabidopsis* cultured cells at various simulated gravity levels. Considering that the duration of the eukaryotic cell cycle is approximately 24 hours^21,22^, our experiments consisted of a series of treatments of different duration involving the exposure to altered gravity conditions for 3, 14, and 24 hours. We found that the proportion of cells in the different cell cycle phases was changed by exposures of the various durations at different (simulated) gravity levels (Fig. 1). After 3 hours, the distribution of the cell cycle phases among different gravity levels showed no significant alterations. Only in the sample simulating Mars gravity a small increment on the proportion of cells in S-phase was observed (Fig. 1A). However, after 14 hours, the number of cells in S-phase increased significantly in all conditions of altered gravity, accompanied by a reduction of the proportion of cells in G1, especially under simulated microgravity and hypergravity (Fig. 1B). The 24-hour experiment showed more-defined influence of the change in the gravity levels on the cell cycle phase distribution. Both simulated reduced gravity samples showed the same trend, producing a significant increment on the G2/M and the S-phase rates at the expense of the G1 rate (Fig. 1C). Also, the proportion of cells in G1 under simulated microgravity and simulated Mars gravity was reduced more as the exposure duration increased. In the case of the 24-hour experiment, this reduction in G1 cell proportion was statistically significant in these conditions, compared to the 1 *g* control (Fig. 1C). Regarding the hypergravity treatment, it was noted a significant reduction of S-phase cells after 24 hours of exposure (Fig. 1C).

### Simulated reduced gravity levels increased the subpopulation of cells replicating DNA: detection of S-phase cells using EdU labeling assay

**Figure 2:**
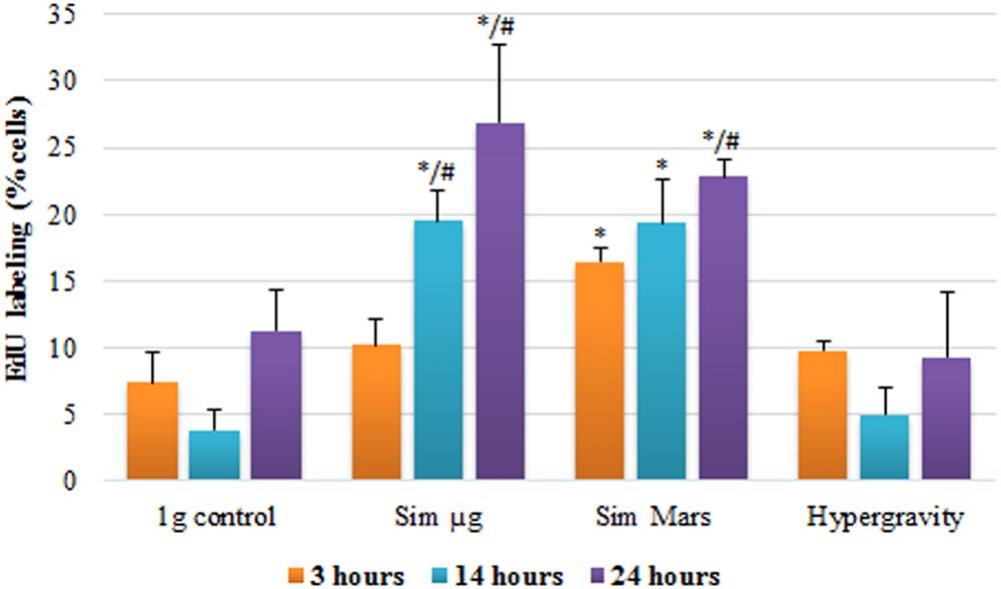
Direct determination by EdU labeling of the S-phase subpopulation of the cell culture after 3, 14, and 24 hour of exposur to different conditions of gravity. Percentage of cells labeled in D plot obtained from bi-parametric dot-plot analysis (not shown). Cells were exposed to different altered gravity levels: simulated microgravity (Sim µ*g*), simulated Mars gravity, and 2g hypergravity, for experimental durations of 3, 14 and 24 hours. 10000 cells in three replicates were counted by flow cytometry. The results were compared to the 1 *g* control. Data shown mean ± SE of the three replicates. Significant variations by gravity level *versus* 1 *g* control at the same exposure experiment (*), and by exposure time *versus* the same gravity level at 3 hour experiment (#) are shown. (P-value, * and ^#^ < 0.05).

EdU (5-ethynyl-2′deoxyuridine) labeling assay is a rapid and robust approach for detection of the S-phase (DNA replication phase) of the cell cycle and, indirectly, to evaluate the cell proliferation rate^42^. This assay is a better alternative than the indirect flow cytometry determination of DNA content after DAPI staining in which the proportion of cells in S-phase was obtained by exclusion of the cells in G1 and the cells in G2/M periods from the total cell population, as shown in the preceding section. The EdU assay showed an increase in the S-phase subpopulation of cells through the exposure time at the two levels of simulated reduced gravity (simulated microgravity and simulated Mars gravity), as expressed in the percentage of cells under replication (Fig. 2). After 3 hours of exposure, the percentage of labeled cells was significantly increased only under simulated Mars gravity. Increasing the exposure time (14- and 24-hour experiments), the differences with the 1 *g* control became statistically significant (p < 0.05) in all the reduced gravity conditions (Fig. 2). Hypergravity samples showed little variations with the 1 *g* control on the number of replicating cells at any time of exposure, although a reduction on the percentage of the labeled cells was observed with the progression of the exposure time, at 14 and 24 hours (Fig. 2).

### The gravitational alteration influenced nucleolus structure and activity

**Figure 3:**
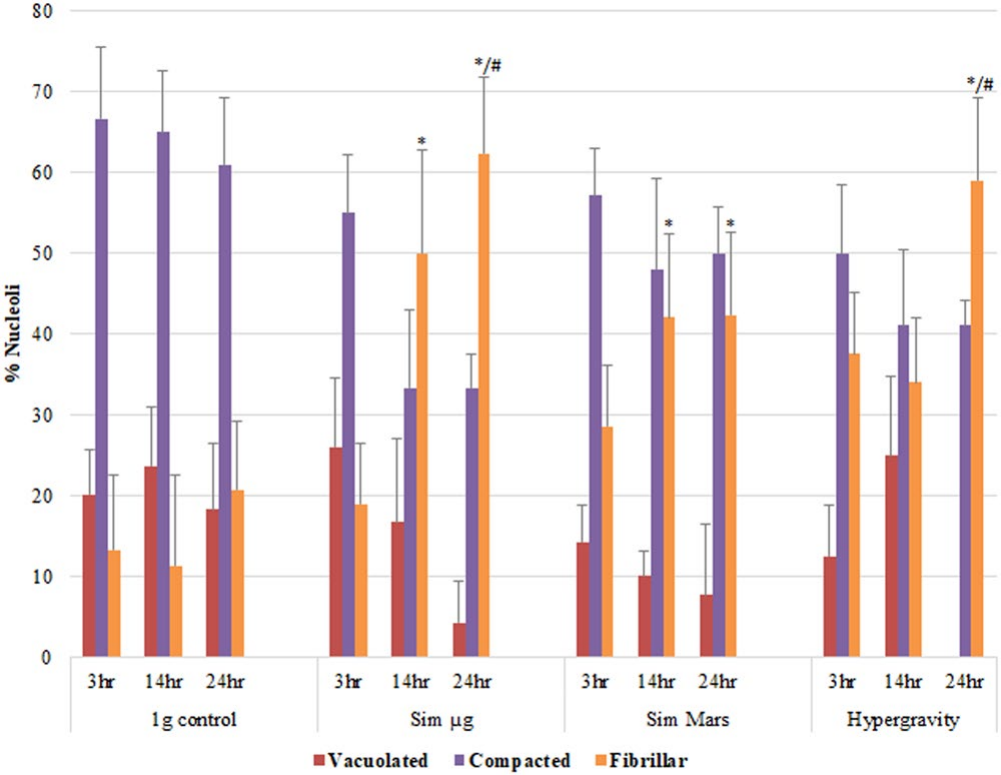
Effect of gravity alteration on the distribution of nucleolar structural types for different exposure times (3, 14, and 24 hours). Various gravitational alterations, namely simulated microgravity (Sim µ*g*), simulated Mars gravity and 2g hypergravity, induce modifications in the distribution of the nucleolar structural models. The different morphofunctional types of nucleoli, namely vacuolated, compact and fibrillar (arranged according to their activity from highest to lowest), as defined by Manzano^11^, were identified using phase contrast microscopy (see Supplementary Figures 2,3 and 4). On average, 10 cells were counted per conditions for 5 technical replicates. Data shown mean ± SE (error bars). Significant variations by gravity level *versus* 1 *g* control at the same exposure experiment (*), and by exposure time *versus* the same gravity level at 3 hour experiment (#) are shown are shown. (P-value, * and ^#^ < 0.05).

In highly proliferating cells, the structural parameters of the nucleolus represent a good estimation of the rate of ribosome biogenesis and, consequently, of the level of protein synthesis and the cell growth^43^. In fact, the nucleolus and nucleolar activity have been identified as efficient and reliable indicators of cellular stress^44,45^, and, in the case of the gravity alterations, the exposure of *Arabidopsis* cell cultures at various gravity levels produced variations on the nucleolus structure, which can be interpreted with functional criteria^11,46^. Thus, we counted the three different structural models of the nucleolus which can be identified in a population of proliferating cells, representing morpho-functional models differing in structure and nucleolar activity^11^. The three structural types found in *Arabidopsis* proliferating cell cultures were vacuolated, compact, and fibrillar nucleoli, listed in decreasing order of activity (Supplementary Figures 2,3 and 4). The relative proportion of the three nucleolar structural types was shown to represent an accurate estimation of the rate of ribosome biogenesis and hence of cell growth^11^. Our estimation of the distribution of these morpho-functional types revealed that exposing cells to gravitational alteration led to a reduction of nucleolar activity through the exposure time (Fig. 3, and Supplementary Figures 2,3 and 4).

In the 3-hour experiment, the compact nucleolus was the major structural model found in the cell population under all gravity levels, as it occurs in 1 *g* control cells. In general, fibrillar nucleoli “inactive nucleoli” population increased significantly under all gravitational conditions as the duration of the treatment increased. This effect was clear for the simulated microgravity treatment, and it was maximized after the 24-hour exposure when more than half of nucleoli showed a fibrillar/inactive structure with almost no vacuolated nucleoli in altered gravity samples, whatever the level of gravity simulated (Fig. 3).

### Gravitational alterations reduced ribosome biogenesis regulatory proteins

**Figure 4:**
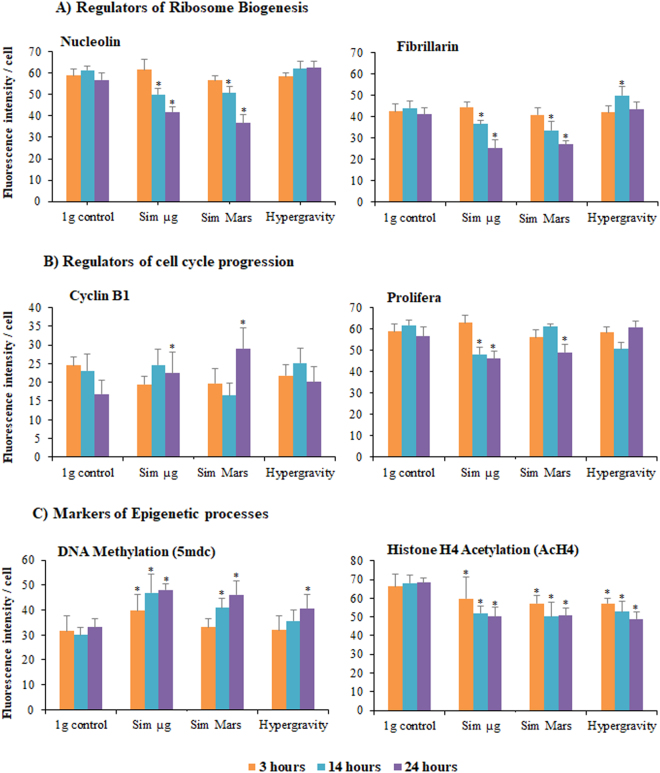
Effect of altered gravity on the levels of relevant proteins, used as markers of cellular functions related to cell proliferation and cell growth. Mean fluorescence intensity per cell measured by flow cytometry, using specific antibodies revealed by specific fluorescent secondary antibodies. Selected proteins were: (**A**) Regulators of ribosome biogenesis acting at different levels of pre-rRNA synthesis and processing (Nucleolin and Fibrillarin); (**B**) Regulators of cell cycle progression, acting at different cell cycle checkpoints (CyclinB1 and Prolifera), and (**C**) Epigenetic markers of DNA methylation (5-methyl-deoxycytidine; 5mdc) and Histone H4 Acetylation (Acetyl-histone H4; AcH4). Cells were exposed to different gravity levels: 1 *g* control, simulated microgravity (Sim µ*g*), simulated Mars gravity, and 2g hypergravity for three durations: 3, 14, and 24 hour. Fluorescence intensity per cell was quantified using flow cytometry on 10,000 cells labeled with each specific antibody. Data shown mean ± SE of the three replicates. Significant differences with 1 *g* control are indicated (*). P-value < 0.05.

We have selected two proteins as accurate markers of the cellular functional processes related to cell proliferation and cell growth, and we have quantitatively evaluated variations in their levels induced by altered gravity using immunofluorescence and flow cytometry analysis. Alterations on the ribosome biogenesis function have been assessed using specific antibodies to detect nucleolin (AtNUC1) and fibrillarin (AtFIB1) proteins. Three hours of exposure was not enough to alter the nucleolin fluorescence intensity in any gravity level (Fig. 4A). However, increasing the exposure time to 14 hours resulted in a significant depletion of the nucleolin expression in reduced gravity levels (simulated microgravity and simulated Mars gravity). This effect became significantly greater (p < 0.05) after 24 hours of exposure. On the contrary, hypergravity showed no statistically significant differences compared with the 1 g control (Fig. 4A).

Like nucleolin, fibrillarin is an important regulator of the first steps of pre-rRNA processing and hence of the production of ribosomes. Simulated reduced gravity conditions showed a significant reduction of the fibrillarin levels after the 14-hour exposure, which was stronger after the 24-hour treatment. Hypergravity conditions enhanced the cellular amount of fibrillarin, reaching significance after 14 hours, but not after the 24-hour experiment (Fig. 4A).

### Cell-cycle regulatory proteins were affected by the gravitational alterations

After having found changes in the duration of *Arabidopsis* cell cycle phases caused by altered gravity levels, we have searched for alterations of regulatory mechanisms of the cell cycle, which could give account of these changes. For this study, we have chosen Cyclin B1, which plays a role in the regulation of G2/M checkpoint^14,19^ and the antigen called Prolifera, whose regulatory activity takes place in the G1/S checkpoint^47,48^. The cellular amounts of these two proteins were recorded by measuring the immunofluorescence intensity after flow cytometry.

Exposure for 3 hours was not enough to produce significant changes in the levels of the two proteins. A significant increase of the level of Cyclin B1 under the simulated Mars gravity was detected in the 24-hour treatment, but not after 14 hours. Also, simulated microgravity showed a significant increment in the levels of Cyclin B1 in the 24-hour treatment. Hypergravity treatment did not appear to produce significant effects (Fig. 4B).

In contrast, the fluorescence intensity of Prolifera decreased in simulated microgravity after the 14- and 24-hour treatments and, after 24 hours of exposure, in the simulated Mars gravity. In turn, hypergravity showed low influence on the Prolifera levels (Fig. 4B).

### Gravitational alterations impacted epigenetic mechanisms

Methylcytidine base in DNA was detected using the same flow cytometry approach as used to evaluate protein levels (anti-5mdc antibody). Hypermethylation pattern increased with exposures to all altered gravity conditions tested and the intensity of the effect was higher in long-duration treatments. Exposure for 3 hours was enough to show alterations in DNA methylation only under simulated microgravity; however, increased exposure times up to 14 hours induced a statistically significant (p < 0.05) hypermethylation under the two simulated reduced gravity levels, which was confirmed in the samples exposed for 24 hours. Hypergravity samples showed the same pattern only after 24 hours of exposure (Fig. 4C).

Histone acetylation, as determined by flow cytometry with an anti-AcH4 antibody, was quickly modified by altered gravity conditions, showing a significant depletion in the 3-hour treatment. In general, these depletions were intensified at the 14-hour and maintained at the 24-hour exposures. This occurred in all the altered gravity simulations, in this case including 2g hypergravity (Fig. 4C).

### Gravitational alterations produced changes at the gene transcription level

**Figure 5:**
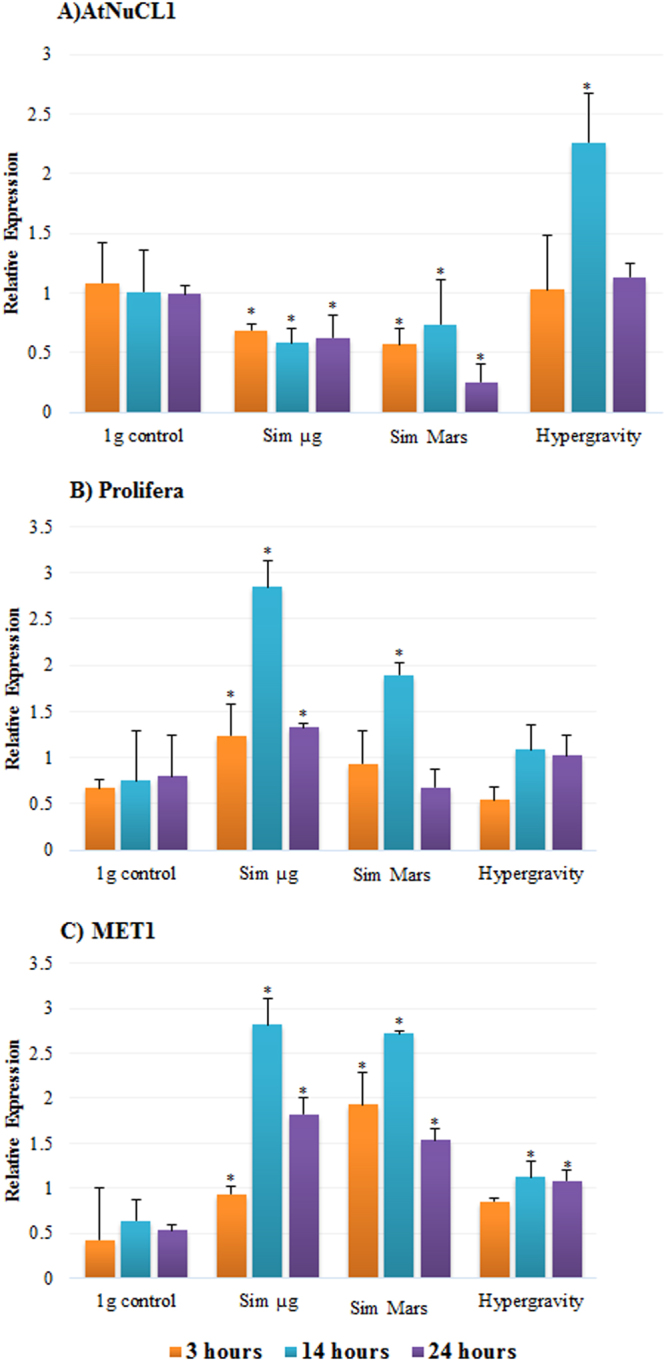
Changes in the transcription of genes induced by altered gravity levels, estimated by qPCR. The selected genes, taken as markers of cellular processes, are as follows: AtNuc1/nucleolin (ribosome biogenesis), Prolifera (cell proliferation), and MET1/DNA Methyltransferase (epigenetic mechanisms /DNA methylation). Cells were exposed to different altered gravity levels: simulated microgravity (Sim µ*g)*, simulated Mars gravity, and 2g hypergravity, for three durations: 3, 14, and 24 hour, and compared to the 1 *g* control. Relative gene transcription was evaluated using qPCR analysis, relative to the transcription levels of the *Actin* reference gene (Supplementary Figure 5). Data shown mean ± SE of the three replicates. Significant differences in comparison with 1 *g* control in the same experimental conditions are shown (*). P-value < 0.05.

We have also analyzed the impact of gravity alteration on the same essential cellular processes at the gene transcription level. We have used genes coding for some of the proteins that were analyzed in their levels by flow cytometry, whose transcription was quantitatively assessed by qPCR (Fig. 5). In particular, we have estimated changes induced by our simulations on the transcription levels of AtNUC1 (nucleolin 1) gene, taken as marker of ribosome biogenesis, the Prolifera gene, taken as marker of cell proliferation, and MET1 (DNA (cytosine-5)-methyltransferase 1) gene, taken as marker of epigenetic mechanisms. Nucleolin transcription, as a cell-growth-related marker, showed a general decrease in the two levels of simulated reduced gravity, and in the three experiments using different times of exposure. The highest reduction in the transcription level was recorded after 24 hours of exposure to the simulated Mars gravity. On the contrary, hypergravity induced higher nucleolin transcription levels, particularly significant after 14 hours of exposure (Fig. 5A). Furthermore, the Prolifera gene transcription level was rapidly increased under simulated microgravity. Statistical significance (p < 0.05) was reached just at the 3-hour exposure and enhanced after 14 hours. Similar results were obtained for simulated Mars gravity. It is noticeable that, for the two simulated reduced gravity treatments, the highest differences with the 1 *g* control have been achieved in the 14-hour exposure experiments. In turn, the Prolifera gene transcription level was not affected by hypergravity (Fig. 5B). Transcription of the marker of epigenetic mechanisms, namely DNA Methyltransferase (MET1), was increased significantly under simulated reduced gravity levels. Like in the previous case, the alterations in the transcription of this gene for the two conditions peaked at the 14-hour experiments and were later reduced in 24-hour exposures. A weaker effect was observed in the hypergravity, in which the significant alteration occurred after 14- and 24-hour exposures (Fig. 5C).

### Co-localization of epigenetic mechanisms under the gravitational alterations effect: DNA methylation and histone H4 acetylation activities: Analysis of multicolor confocal immunofluorescence microscopy images

**Figure 6:**
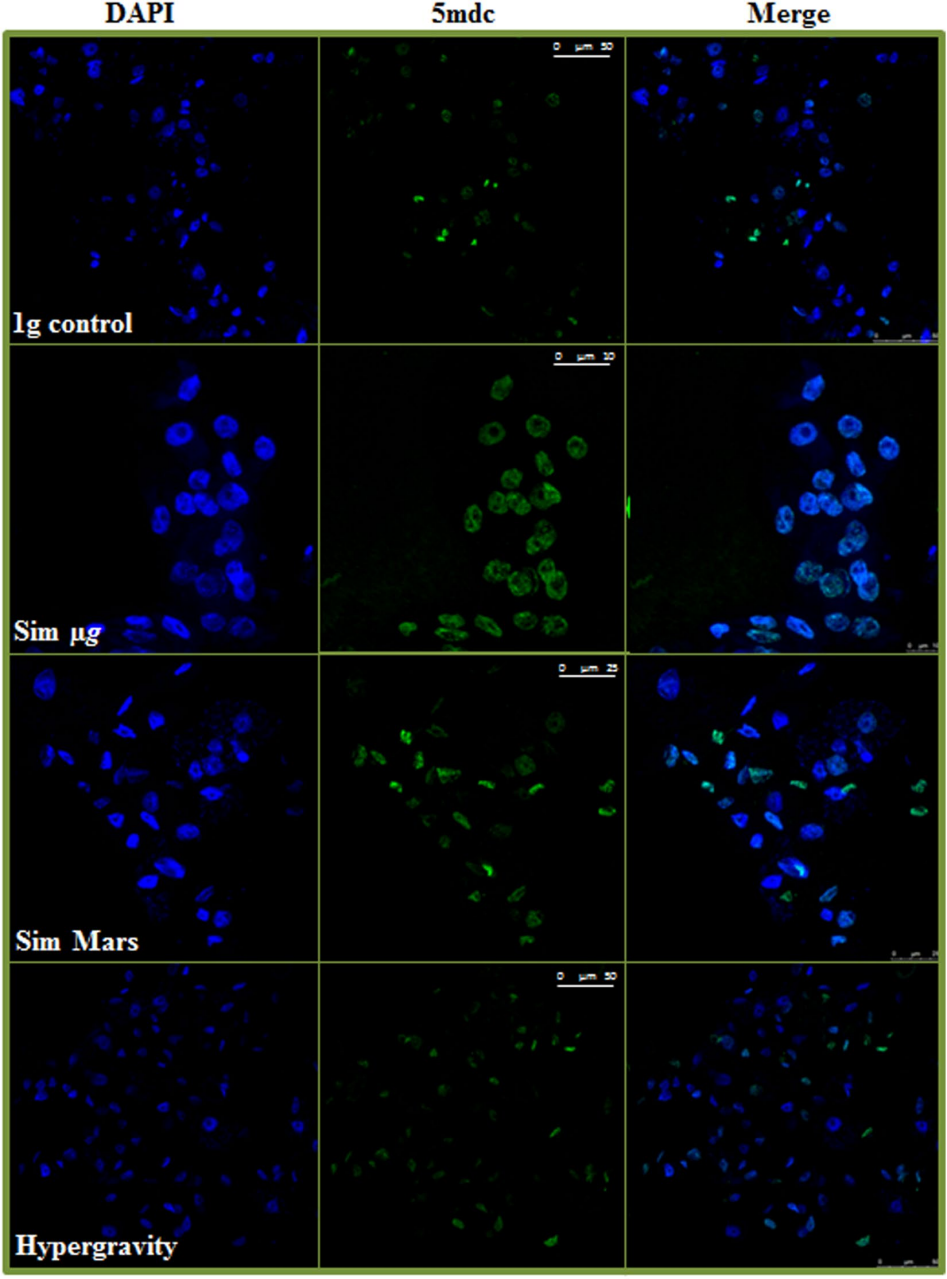
*In situ* patterns of DNA methylation (5MdC) under different gravity conditions, detected by immunofluorescence. Cells were exposed to 1 g control gravity and different gravitational alterations, namely simulated microgravity (Sim µg), simulated Mars gravity, and 2g hypergravity for 14 hours of exposure. Confocal images show immunolocalization of 5MdC (green) and the chromatin pattern (DAPI). The merged images show the methylation pattern in the spatial context of nuclear DNA. The increase of methylation in samples exposed to simulated microgravity and, in a lesser extent, in samples grown under simulated Mars gravity, compared to the 1 *g* control, is shown. Hypergravity does not appear to produce relevant changes. Magnification bar affects to the three images of the same microscopic field and gravitational.

**Figure 7:**
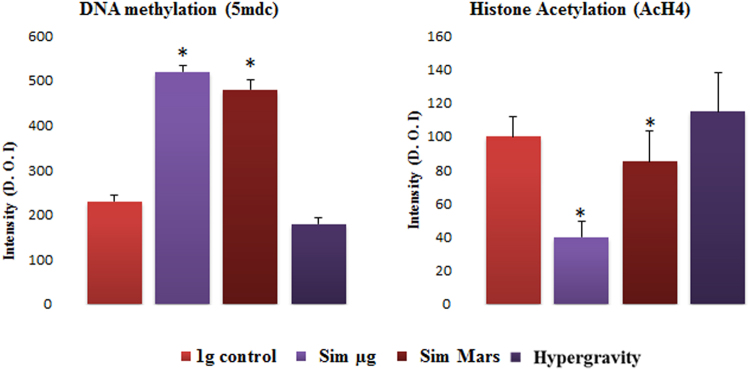
Quantitative study of the changes induced by altered gravity on DNA methylation (5MdC) and Histone H4 acetylation (AcH4) measured by the fluorescence intensity levels after immunolocalization. Cells were exposed to 1 *g* control gravity and different gravitational alterations, namely simulated microgravity (Sim µ*g*), simulated Mars gravity, and 2g hypergravity for 14 hours of exposure. Quantitative fluorescence intensity level was determined using Leica confocal software in individual cells to detect the green signal intensity. An average of 100 cells were measured in three different biological replicates. Data shown mean ± SE of the three replicates. Significant differences in comparison with 1 *g* control are shown (*). P-value < 0.05.

The pattern of nuclear-methylated cytidines was determined by immunolocalization of the distribution of 5-methyldeoxycytidine (5MdC) in genomic DNA. We decided to define the pattern of methylated cytidine in the samples with the most robust results (14-hour exposure) (Fig. 6). Cells exposed to simulated reduced gravity were shown to contain larger and more abundant fluorescent spots, indicating a higher presence of 5MdC in nuclei. This alteration was maximized under simulated microgravity conditions, while 2g hypergravity did not show significant changes on the distribution of 5MdC. This observation was supported by the immunofluorescence quantification, in which simulated reduced gravity levels (simulated microgravity and simulated Mars gravity) showed significantly higher levels of 5MdC intensity (Fig. 7).

**Figure 8:**
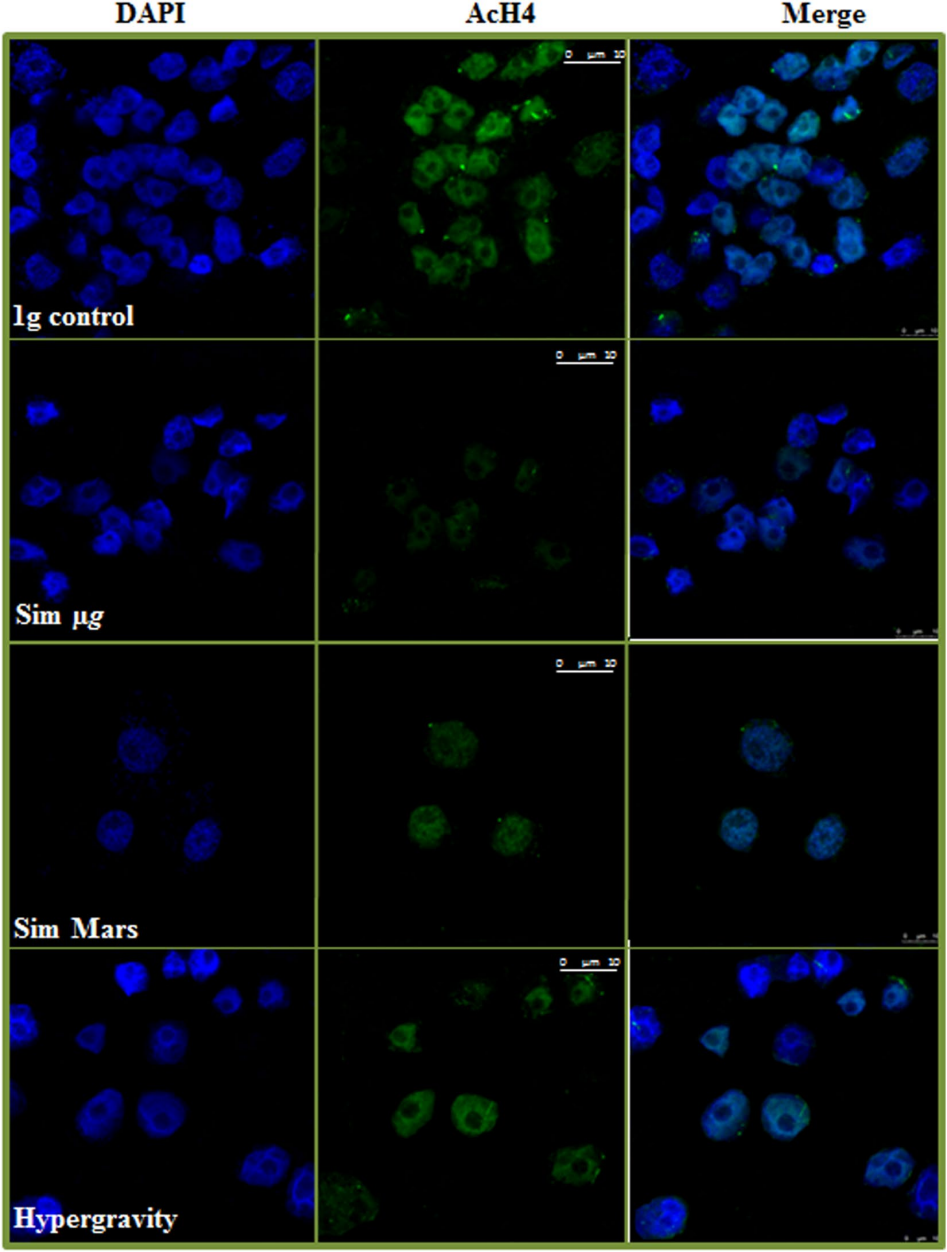
*In situ* patterns of Histone H4 Acetylation (AcH4) under different gravity conditions, detected by immunofluorescence. Cells were exposed to 1 *g* control gravity and different gravitational alterations, namely simulated microgravity (Sim µ*g*), simulated Mars gravity, and 2g hypergravity for 14 hours of exposure. Confocal images show immunolocalization of histone acetylation (green) and the chromatin pattern (DAPI). The merged images show the acetylation pattern in the spatial context of nuclear DNA. The decrease of acetylation in samples exposed to simulated microgravity and, in a lesser extent, in samples grown under simulated Mars gravity, compared to the 1 g control, is shown. Hypergravity does not appear to produce relevant changes. Magnification bar affects to the three images of the same microscopic field and gravitational.

In parallel, the distribution pattern of the histone H4 acetylation (immunolocalization of AcH4) was shown to be modified by the gravitational alteration in comparison with the control conditions after 14 hours of exposure to experimental conditions (Fig. 8). It was observed that AcH4 immunofluorescence pattern was different from the 5MdC pattern after the influence of the various gravity levels. The presence of AcH4 in the chromatin was significantly reduced under simulated reduced gravity being severely depleted under simulated microgravity, while hypergravity showed no significant changes. The quantitative analyses of the immunofluorescence intensity supported the reported observations on the presence of AcH4 after the gravitational alterations. Results revealed that AcH4 immunofluorescence intensity level was depleted under simulated reduced gravity, even though the difference was not statistically significant under the simulated Mars gravity. On the other hand, the impact of hypergravity was not significant enough to alter the AcH4 intensity (Fig. 7).

## Discussion

In this study, we report changes on cell cycle and cell growth using an *in vitro* plant cell culture, induced by altered gravity simulated by ground-based facilities (GBF), namely simulated microgravity, simulated partial gravity (Mars gravity) and hypergravity (2g). The results obtained in GBFs in our model system provide evidence of a cellular response to an altered perception of the magnitude of the gravity vector. This response can be put in relation to the response of meristematic cells exposed to real or simulated microgravity and provides new parameters and new insights for the discrimination of the specific mechanisms of response at the cellular level. Moreover, we have analyzed epigenetic mechanisms involved in these changes induced by altered gravity, which have a significant influence on the processes of plant growth and development^1,2,49^.

Our experiments included exposures of different durations in order to identify the minimum duration of the environmental disturbances capable of affecting the functions studied in the cell culture. Taking into account the length of the eukaryotic cell cycle^21,22^, it is clear that the treatment of 24 hours corresponds to an entire cell cycle, 14 hours to, approximately, a half, and 3 hours to a fraction of less than 15%. The existence of these effects, even at the shortest of these treatments, indicates a high sensitivity of the regulatory mechanisms to the altered gravity simulated conditions. The inclusion of a single cell cycle checkpoint within the treatment is enough to produce detectable effects. However, the correlation of the intensity of effects with the duration of the treatment indicates that not all the cell cycle phases are equally sensitive to the altered gravity treatment. Most probably, the effects of the treatment only (or mostly) occur when it includes one or more of the known checkpoints (G1/S, G2/M, and anaphase).

The progression of the cell cycle is disrupted in response to the effective gravitational load. Cell cycle phase distributions changed after 14 and 24 hour exposures to simulated altered gravity. Results obtained from the flow cytometry study on cells exposed to simulated reduced gravity (micro- and Mars gravity levels) revealed that cells are accumulated in S-phase, gradually passing to the G2 period, and then to mitosis, through the exposure time. Furthermore, a reduction on the proportion of cells in G1 is observed, whose meaning is that cells are entering faster into the S-phase. EdU labeling confirmed accumulation of cells in S-phase. Also, the proportion of cells in G2 also showed an increment under simulated reduced gravity conditions. In contrast, the cell cycle shows a slower progression under hypergravity, resulting in a lower rate of cell proliferation. The acceleration of the cell cycle is the consequence of changes in cell cycle regulators, such as cyclin B1, detectable at the levels of both gene transcription and cellular protein amount. The enhancement of the cell proliferation rate and the change in the cyclin B1 gene transcription levels were reported in *Arabidopsis* root meristematic cells of seedlings grown under real or simulated microgravity^8–10^ and in semi-solid cell cultures^11^. Otherwise, the acceleration of the cell cycle is well-matched with a higher level of the Prolifera antigen (both at the protein and transcriptional level) under simulated reduced gravity levels. Prolifera is a minichromosome maintenance (MCM) protein complex which enters into the nucleus, binds chromatin and participates in the initiation of replication^50^ suggesting that it plays a key regulatory role in G1/S transition^47,48^.

Regulation of ribosome biogenesis is linked to factors controlling cell growth and proliferation^28^. Specifically, in cellular systems programmed for proliferation, such as the suspension cell culture or the root meristem, the concept of “cell growth” unequivocally means the increase in cellular biomass necessary to reach a certain threshold enabling cell division and the viability of the daughter cells. In this case, this increase of biomass is produced at the expense of protein biosynthesis and, therefore, it is correlated with the rate of production of ribosomes. Ultimately, cell growth in these cellular systems depends on the activity of RNA polymerase I in the transcription of pre-rRNA^24,25^. This concept of “cell growth” should be distinguished from other processes producing an increase of the cell size at the expense of the formation of vacuoles, usually associated with cell differentiation, which should be appropriately called “cell expansion”^23,26,27,51^. In our experiments the rate of ribosome biogenesis has been quantitatively assessed by using the nucleolar proteins nucleolin^52,53^ and fibrillarin^54–56^ as markers of ribosome biogenesis, whose levels are known to correlate to the rate of production of ribosomes. Both transcript and protein expressions of these markers were measured. Nucleolin and fibrillarin protein levels decreased with the exposure to simulated reduced gravity, while the alterations caused by hypergravity were not evident. Moreover, the alteration on nucleolin protein expression is stronger than in the case of fibrillarin, which has a more accurate role in the early steps of pre-rRNA processing. The nucleolin protein decrease is supported by the significant down-regulation of the *AtNUC1* gene transcription. Additionally, the alteration in gene transcription was quick, even in the 3-hour experiment, compared with the reduction in the protein levels, which reached significance only after 14 hours under simulated microgravity.

Data obtained on regulation of ribosome biogenesis are in complete agreement with the results on the morpho-functional features of the nucleolus, a highly polymorphic structure, which changes its structure in response to functional alterations, as it has been reported by numerous investigators^57^. It is well established that the rate of ribosome biogenesis can be estimated throughout the molecular cytology of the nucleolus^28,58^. The nucleolar morpho-functional types, which were identified and characterized using the electron microscope^11,59^ could be easily identified using phase-contrast microscopy in samples exposed to differential gravitational effects. Under both simulated reducedd gravity and hypergravity, there was a steady significant decrease in the number vacuolated and compact nucleoli cells (those types characterized by a higher activity), at the expenses of an increase in the inactive fibrillar nucleoli, which was maximized after the 24 hour experiment with the complete loss of vacuolated nucleoli models. These results are entirely consistent with previous results of our laboratory using *Arabidopsis* semisolid cell cultures under simulated microgravity^11^ and root meristems under real and simulated microgravity^10^.

Epigenetic mechanisms in the chromatin can be considered as a plant systemic response to gravitational alterations. The regulation of genes via cytosine methylation and histone modification is a well-recognized component of the plant responses to environmental stresses^60,61^. In our *Arabidopsis* cell cultures, an increase in the overall cytosine methylation pattern was detected under simulated reduced gravity conditions. The results of DNA methylation, based on immunofluorescence protein assays (flow cytometry and confocal microscopy) showed a quick and extensive hypermethylation of cytosine residues through different times of exposure to simulated reduced gravity conditions, including simulated microgravity and simulated Mars gravity. This observation is consistent with the up-regulation of DNA methyltransferase (MET1) gene transcription quantitatively detected by qPCR. This enzyme is mainly involved in maintaining symmetric cytosine methylation, suggesting that DNA methylation could participate in the regulation of gene transcription in response to the gravitational stress. Similarly, we analyzed histone acetylation. The presence of the acetylated histone H4 appeared increasingly depleted by the simulated reduced gravity treatments through the exposure time, thus indicating that reduced gravity load causes histone deacetylation. Some rice and *Arabidopsis* studies shown that histone deacetylation is involved in ABA and stress response pathways^62,63^.

In conclusion, our results suggest that different altered gravity environments produce a serious stress on proliferating plant cells, capable of uncoupling cell proliferation and cellular growth. The use of simulation facilities in partial gravity studies is promising, but it should be further tested and validated *versus* real flight data in the future in order to confirm or reject the user potential of RPM for microgravity and partial g simulator. To determine effective countermeasures for the gravitational stress and the mechanisms and strategies for the plant adaptation and survival in the altered gravity environment is one of the greatest challenges of space plant biology with significant general consequences for plant physiology and cell biology.

## Materials and Methods

### Immobilization of cell suspension culture

We adopted a procedure for immobilizing cells in agarose designed to be used in spaceflight^64,65^. The full technical details and validation of the modified procedure used are found in a previous publication^4,66^. Briefly, *Arabidopsis* cell suspension culture (MM2d**)** was grown in Murashige and Skoog medium (MSS) shaking at 120 rpm at 27 °C in the dark^17^. MM2d culture was embedded in low melting agarose (1% (w/v) and cells, either fixed or frozen, were recoved at the end of each experiment. Agarose-embedded cells were fixed by adding 1 ml of 4% (*w/v*) paraformaldehyde (PFA, Electron Microscopy Sciences, Hatfield, PA, USA) in phosphate-buffered saline (PBS buffer) onto the surface of the plate for 1 hour and recovered by centrifugation after dissolving the agarose by immersion in a water bath at 63 °C. Samples to be recovered frozen were first gently fixed with 1 ml 1% (*w/v*) PFA for 15 min, to arrest the biological activity. Then, cells were successively dissolved in a water bath, concentrated by centrifugation and directly frozen by immersion in liquid nitrogen. The time needed to collect frozen samples was always less than 1 hour after the end of the experiment.

### Experimental design

Agarose-embedded *Arabidopsis* cell suspension cultures were used to study the impact of altered gravity levels. To reproduce the effects of altered gravity, two devices were used (Supplementary Figures 1); the random positioning machine (RPM: simulated microgravity and simulated partial gravity (Mars gravity) and the Large Diameter Centrifuge (LDC: hypergravity). The 1 *g* control experiment was performed with the samples attached to the RPM frames, in order for the control cells to be subjected to the same temperature and as closely as possible to any residual vibrations for samples grown in the rotating RPM. To reduce the extracellular fluid motion effect to a minimum, the cells are immobilized in agarose^65,66^. The RPM in its original configuration is a widely used simulator for microgravity, particularly validated in the use with plant systems^3,67^. To produce simulated microgravity, a real random algorithm was used with the maximum angular speed set at 60° per second with random direction and interval. To produce simulated fractional gravity of Mars (0.37 g), a novel algorithm driving the RPM motion, extensively described and validated elsewhere^68^ was used. In short simulated microgravity is provided by a standard RPM, the randomness of the algorithm is such that over time an imaginary sphere would be perfectly covered with the random paths of the two frames of the RPM would describe, time averaging the g vector orientation^5^. The novel algorithm we used in this study for Mars partial g simulation is such that the shape of the imaginary sphere is deformed to a prolate spheroid, the shape of which defined the skewed orientation of the gravity vector such that there is, over time, an averaged preferred orientation towards to Earth gravity field resulting in a partial randomization of the g vector, so a certain level of partial gravity remains present (in our case 0.37 g). Hypergravity (2g) environment was obtained using the LDC^7^. We decided to use 2g as a moderate increased gravitational stimulus, potentially enough to activate the specific response to increased load, and moderate enough not to compromise cell integrity and saturate global stress pathways. The study was performed at the European Space Technology Center (ESTEC) of the European Space Agency (ESA) in Noordwijk, The Netherlands. Experiments were performed for different times of exposure to altered gravity: 3, 14, and 24 hour), each in three replicates, at 27 °C, in the dark.

### Flow cytometry and cell cycle analyses

Each sample of frozen cell pellet (500 mg) was treated with the High-Resolution Kit for plant ploidy level analysis [Kit Cystain UV precise type P, containing solution A (Nuclei extraction buffer) and solution B (Staining buffer containing DAPI -4,6,diamino-2-phenylindol), Partec GmbH, Munster, Germany] to determine the DNA content. To release cell nuclei, we rinsed the cells in solution A and carefully chopped with a sharp razor blade. Then, cells were filtered and rinsed in solution B^17^. On average, 10000 particles in three replicates were counted by flow cytometry (Cell Sorter FACS Vantage, Becton–Dickinson, San Diego, California, USA), using ion laser tuned at 360 nm and detection of emission using a blue fluorescence emission filter (bandpass filter of 424/44 nm). FACS analysis results were analyzed using BD CellQuest™ software to determine the ratios of cell cycle phases per the DNA content of individual cells (2n for phase G1, 2 < n < 4 for S, 4n for G2/M phases).

### EdU-based proliferation assay

EdU (5-ethynyl-2′-deoxyuridine) labeling is a method for detecting and quantifying newly synthesized DNA. EdU is a nucleoside analog of thymidine and is incorporated into DNA during active DNA synthesis. EdU assay protocol for *Arabidopsis* cell proliferation studies was followed and adapted^4^ from Kotogany and colleagues^42^. One mL of 10 µM EdU in DMSO (Invitrogen, Click-iT EdU Alexa Fluor 488 HCS assay, *cat no: A10027*) was added onto the surface of the experimental Petri dishes (cells embedded in agarose) 2 hours before the end of each experiment and 0.1% DMSO was used as a control for the EdU-based assay test. Cells were directly frozen at the end of the experiment and then processed for nuclei isolation protocol. Isolated nuclei were fixed for 15 min in 1% (w/v) paraformaldehyde solution in PBS with 0.1% Triton X-100. Adding the detergent Triton X-100 in the fixer prevents cell shrinkage and it also partially permeabilizes the plasma membranes for small detection reagents required in the EdU assay. Following 3 × 5 min PBS washes. 20–30 μl packed cell volume of cells were directly incubated 30 min at RT in EdU detection cocktail. For 1 sample reaction, following amounts of the kit components are mixed in 144 μl distilled water; 1.6 μl buffer additive (component F, kept frozen in small aliquots), 14 μl reaction buffer (Component D), 6.7 μl Copper (II) sulfate solution (Component E, 100 mM CuSO4) and 0.07 μl Alexa Fluor 488 azide (Component B, in 70 μl DMSO). The detection cocktail should be prepared freshly. Although the click reaction is not light sensitive, fluorochrome containing solutions should not be exposed to strong light. Nuclei were stained with 100 ng/ml DAPI and analyzed on a FACS cell sorter. On average, 10000 cells in three replicates were counted by flow cytometry approach. Two fluorescence detectors were used: with the standard 480 nm laser, Alexa Fluor 488-EdU intensity was detected between 495–519 nm, whereas for detection of DAPI (4′,6-diamidino-2-phenylindole) intensity (DNA content), 355–460 nm emission range was used under 360 nm UV laser.

### Specific protein quantification in single cells using flow cytometry

Flow cytometry quantified the levels of proteins labeled by immunofluorescence by adapting the method previously used in our laboratory for isolated onion cell nuclei^4,69^. Isolated nuclei were filtered on ice and incubated in isolation buffer for 2 min. After centrifugation, they were fixed in 1% (*w/v*) PFA in PBS, for 15 min, on ice. Fixed nuclei were washed twice with 0.01% (*v/v*) Triton X-100 in PBS by centrifugation at 4 °C (5 min, 1500 rpm). Stained nuclei were incubated in 500 μl of blocking solution (2% (*w/v*) BSA and 0.05% (*v/v*) Tween in PBS) for 30 min at 4 °C. Following centrifugation at 4 °C (5 min, 1500 rpm), cells were incubated with the first antibody in blocking solution for 45 min at 4 °C (see antibodies used and their corresponding dilutions in Supplementary Table 1). Then cells were washed again with PBS (2 × 5 min), using centrifugations at 1500 rpm and incubated in Alexa Fluor®-labeled secondary antibody (Supplementary Table 1) for 30 min at 4 °C. After a new wash with PBS (3 × 5 min), nuclei were counterstained with the staining solution B, containing DAPI. Two fluorescence detectors were used with the standard 360 nm laser: Alexa Fluor®-protein fluorescence intensity was detected between 495/670 nm, and 355–460 nm emission range was used under 360 nm UV laser for detection of DAPI intensity (DNA content). Fluorescence intensity per cell was quantified using flow cytometry on 10,000 cells labeled with each specific antibody. Data were analyzed using BD CellQuest™ software for fluorescence intensity detected by flow cytometry. Side scatter versus forward scatter diagrams were used to locate and gate nuclear populations by particle size.

### Fixation and processing for immunofluorescence

Supplementary Tabless were additionally fixed in 1 mL of 4% (*w/v*) PFA in PBS, for 1 hour at room temperature (RT) and washed in PBS (3 × 10 min). Then the cell wall was digested using 1 mL of an enzyme cocktail [2% (*w/v*) cellulase, 1% (*w/v*) pectinase, 0.05% (*w/v*) macerozyme, 0.4% (*w/v*) mannitol, 1% (*v/v*) glycerol and 0.2% (*v/v*) Triton X-100] for 30 min at 37 °C. Finally, samples were washed with 1% (*v/v*) glycerol and 0.2% (*v/v*) Triton X-100 in PBS (3 × 10 min). A drop of the cell pellet was placed in a microscope slide covered with poly-lysine and blocked with 2% (*w/v*) BSA and 0.05% (*v/v*) Tween in PBS blocking solution, for 30 min at RT. Samples were incubated with the first antibody (Supplementary Table 1) for 12 h at 37 °C, washed with PBS (3 × 5 min) and incubated with the secondary antibody (Supplementary Table 1), for 3 h at 37 °C, followed by washing with PBS (2 × 5 min) and counterstained with 5 μg/μL DAPI in PBS, for 5 min. After washing with PBS (2 × 5 min) and with distilled water (2 × 5 min), samples were mounted with PVA-DABCO™ (a glycerol based mounting medium containing an anti-fading reagent for use with immunofluorescence preparations) and observed under the confocal microscope (Leica TCS SP5 with AOBS (Acousto Optical Beam Splitter, Mannheim, Germany) with 63× oil immersion optics). Microscopical images were analyzed using the “Leica AF” software (Leica LAS AF v2.4) to estimate the stained nucleolar area.

### Sample processing for ultrastructural analyses

Recovered cells were fixed in 3% (v/v) glutaraldehyde in PBS for 2 hours at RT. Cells were pipetted and rinsed in the fixative during the fixation period. They were then washed 3 times in PBS, 10 min each, dehydrated in an ethanol series, treated for the methylation-acetylation procedure^70^ and finally embedded in LR White resin (London Resin, EMA, UK). From resin-embedded blocks, semi-thin (2 µm thick) sections were visualized by phase contrast microscope (Leica DM2500) and imaged with a Leica DFC320 CCD. The images obtained were processed for quantitative studies using the software programs QWin Standard image analysis (Leica Microsystems) and Image J2.0 (http://www.imagejdev.org). A sample of 10 cells per image were counted for the quantitative studies in three different replicates.

### Quantitative real-time PCR (qPCR)

Total RNA was extracted from frozen samples (0.5 g) using the “REAL Total RNA Spin Plants and Fungi” kit (REAL, Durviz S. L. Lot/21015). It is necessary to ground the cells with liquid nitrogen due to the omnipresence of RNases and to obtain an optimal lysis of the tissues. Extracted RNA was eluted in 30 ml of Nuclease-free water and centrifuge at 13000 rpm for 1 min. And again 30 ml of Nuclease-free water to the same column, with the same collection tube. Centrifuge at 13000 rpm for 1 min. Finally, it will be obtained 60 µl of RNA. RNA concentration and quality were assessed using a spectrophotometer (NanoDrop ND-1000, Thermo, USA). 4 μg of RNA were purified before starting the qPCR using a DNase treatment (Turbo DNA-free; Ambion AM1907). qPCR was performed using DNA amplification and quantification kit (SYBR Green QRT-PCR, Agilent, USA) per the manufacturer, using gene-specific primers (Supplementary Table 2). qPCR running was performed using the iQ™5 Multicolor Real-Time PCR Detection System, Bio-Rad. The qPCR data for each target gene were presented as average transcription levels over three biological replicates, with two technical replicates per reaction, relative to the transcription levels of the *Actin* reference gene. Data analyses were performed using iQ™5 optical system software *v2*.*1*.

### Statistical analyses

Data were collected and analyzed according to Steel^71^. SPSS *v*.*22* software was used to analyze the variance of ifferences using ANOVA test statistically. Degree of freedom was followed as p ≤ 0.05 (95%) considers statistical significance (labeled with an asterisk in figures). Data shown as mean ± SE of different replicates. Standard Error (SE) was estimated using the values of the Standard Deviation (SD) using this formula $({\boldsymbol{SE}}=\frac{{\boldsymbol{SD}}}{\sqrt{{\boldsymbol{n}}}})$.

## Electronic supplementary material

Supplementary information

## References

[1] Herranz R, Medina FJ (2014). Cell proliferation and plant development under novel altered gravity environments. Plant Biology.

[2] Perbal, G. In *A world without gravity* (ed G. Seibert) 121–136 (ESA Publications Division, 2001).

[3] Herranz R (2013). Ground-based facilities for simulation of microgravity, including terminology and organism-specific recommendations for their use. Astrobiology.

[4] Kamal, K. Y. Alterations induced by gravity changes in proliferaing culture cells of Arabidopsis thaliana. *PhD Thesis*, *Universidad Complutense de Madrid*, *Spain* (2014).

[5] Borst AG, van Loon JJWA (2009). Technology and developments for the random positioning machine, RPM. Microgravity Sci. Technol.

[6] van Loon JJWA (2007). Some history and use of the Random Positioning Machine, RPM, in gravity related research. Adv Space Res.

[7] Van Loon, *et al*. The Large Diameter Centrifuge, LDC, for life and physical sciences and technology. *Proc*. *of the ‘Life in Space for Life on Earth Symposium’*, *Angers*, *France*, *22–27 June 2008*. *ESA SP-663*, *December 2008* (2008).

[8] Manzano AI (2013). Meristematic cell proliferation and ribosome biogenesis are decoupled in diamagnetically levitated Arabidopsis seedlings. BMC plant biology.

[9] Matía I (2005). Nucleolar structure and proliferation activity of *Arabidopsis* root cells from seedling germinated on the international space station. Adv Space Res.

[10] Matía I (2010). Plant cell proliferation and growth are altered by microgravity conditions in spaceflight. J Plant Physiol.

[11] Manzano, A. I., Herranz, R., Manzano, A., VanLoon, J. J. W. A. & Medina, F. J. Early effects of altered gravity environments on plant cell growth and cell proliferation: Characterization of morphofunctional nucleolar types in an Arabidopsis cell culture system. F*rontiers in Astronomy and Space Sciences* **3**, 10.3389/fspas.2016.00002 (2016).

[12] Manzano AI (2012). Gravitational and magnetic field variations synergize to cause subtle variations in the global transcriptional state of Arabidopsis *in vitro* callus cultures. BMC Genomics.

[13] Paul AL (2012). Spaceflight transcriptomes: unique responses to a novel environment. Astrobiology.

[14] Menges M, Hennig L, Gruissem W, Murray JA (2002). Cell cycle-regulated gene expression in Arabidopsis. The Journal of biological chemistry.

[15] Gould, A. R. Control of the cell cycle in cultured plant cells. *CRC Crit*. *Rev*. *Plant Sci*. **1** (1984).

[16] Menges M, Murray JA (2002). Synchronous Arabidopsis suspension cultures for analysis of cell-cycle gene activity. The Plant journal: for cell and molecular biology.

[17] Menges M, Murray JA (2006). Synchronization, transformation, and cryopreservation of suspension-cultured cells. Methods Mol Biol.

[18] Mizukami Y (2001). A matter of size: developmental control of organ size in plants. Current opinion in plant biology.

[19] Inze D, De Veylder L (2006). Cell cycle regulation in plant development. Annual review of genetics.

[20] Van Leene J (2010). Targeted interactomics reveals a complex core cell cycle machinery in Arabidopsis thaliana. Molecular systems biology.

[21] Bernard, S. & Herzel, H. Why do cells cycle with a 24 hour period? *Genome informatics**:**International conference on Genome informatics* **17** (2006). 17503357

[22] Cooper, G. M. The EukaryoticCell Cycle. *The Cell: A Molecular Approach*. *2nd edition*. *(Sunderland (MA): Sinauer Associates)*. (2000).

[23] Doerner, P. In *Plant Growth Signaling* Vol. 10 *Plant Cell Monographs (*eds László Bögre & Gerrit Beemster) 1–23 (Springer Berlin/Heidelberg, 2007).

[24] Baserga R (2007). Is cell size important?. Cell Cycle.

[25] Bernstein KA, Bleichert F, Bean JM, Cross FR, Baserga SJ (2007). Ribosome biogenesis is sensed at the start cell cycle checkpoint. Mol. Biol. Cell.

[26] Li C, Potuschak T, Colón-Carmona A, Gutiérrez RA, Doerner P (2005). Arabidopsis TCP20 links regulation of growth and cell division control pathways. Proceedings of the National Academy of Sciences of the United States of America.

[27] Sablowski R, Carnier Dornelas M (2014). Interplay between cell growth and cell cycle in plants. Journal of Experimental Botany.

[28] Sáez-Vásquez, J. & Medina, F. J. Vol. 47 *Advances in Botanical Research* (eds J. C. Kader & M. Delseny) 1–46 (Elsevier, 2008).

[29] Boucheron-Dubuisson E (2016). Functional alterations of root meristematic cells of Arabidopsis thaliana induced by a simulated microgravity environment. Journal of Plant Physiology.

[30] Manzano AI, Herranz R, van Loon JJWA, Medina FJ (2012). A hypergravity environment induced by centrifugation alters plant cell proliferation and growth in an opposite way to microgravity. Microgravity Sci Technol.

[31] Shen-Miller J, Hinchman RR (1995). Nucleolar transformation in plants grown on clinostats. Protoplasma.

[32] Sobol M, Gonzalez-Camacho F, Rodriguez-Vilarino V, Kordyum E, Medina FJ (2006). Subnucleolar location of fibrillarin and NopA64 in Lepidium sativum root meristematic cells is changed in altered gravity. Protoplasma.

[33] Thiel CS (2012). Rapid alterations of cell cycle control proteins in human T lymphocytes in microgravity. Cell Commun Signal.

[34] Barjaktarovic Z (2009). Changes in the effective gravitational field strength affect the state of phosphorylation of stress-related proteins in callus cultures of Arabidopsis thaliana. J Exp Bot.

[35] Kiss JZ (2014). Plant biology in reduced gravity on the Moon and Mars. Plant Biol (Stuttg).

[36] Goldberg AD, Allis CD, Bernstein E (2007). Epigenetics: a landscape takes shape. Cell.

[37] Bernstein BE, Meissner A, Lander ES (2007). The mammalian epigenome. Cell.

[38] Yao B (2016). Epigenetic mechanisms in neurogenesis. Nat Rev Neurosci.

[39] Kouzarides T (2007). Chromatin modifications and their function. Cell.

[40] Costas C, Desvoyes B, Gutierrez C (2011). A chromatin perspective of plant cell cycle progression. Biochim Biophys Acta.

[41] Raynaud, C. *et al*. Chromatin meets the cell cycle. *J Exp Bot*, 10.1093/jxb/ert433 (2014). 10.1093/jxb/ert433 24497647

[42] Kotogany E, Dudits D, Horvath GV, Ayaydin F (2010). A rapid and robust assay for detection of S-phase cell cycle progression in plant cells and tissues by using ethynyl deoxyuridine. Plant methods.

[43] Mélèse T, Xue Z (1995). The nucleolus: an organelle formed by the act of building a ribosome. Current Opinion in Cell Biology.

[44] Mayer C, Grummt I (2005). Cellular stress and nucleolar function. Cell Cycle.

[45] Boulon S, Westman BJ, Hutten S, Boisvert FM, Lamond AI (2010). The nucleolus under stress. Molecular cell.

[46] Manzano, A. I., Herranz, R., van Loon, J. J. W. A. & Medina, F. J. Effects of altered gravity environment on plant cell growth and cell proliferation: Characterization of morphofunctional nucleolar models in an Arabidopsis cell culture system *in vitro* (2014).

[47] Tsuji T, Ficarro SB, Jiang W (2006). Essential role of phosphorylation of MCM2 by Cdc7/Dbf4 in the initiation of DNA replication in mammalian cells. Molecular biology of the cell.

[48] Tanaka S, Tak YS, Araki H (2007). The role of CDK in the initiation step of DNA replication in eukaryotes. Cell division.

[49] Ferl R, Wheeler R, Levine HG, Paul AL (2002). Plants in space. Current opinion in plant biology.

[50] Springer PS, Holding DR, Groover A, Yordan C, Martienssen RA (2000). The essential Mcm7 protein PROLIFERA is localized to the nucleus of dividing cells during the G(1) phase and is required maternally for early Arabidopsis development. Development.

[51] Perrot-Rechenmann C (2010). Cellular Responses to Auxin: Division versus Expansion. Cold Spring Harbor Perspectives in Biology.

[52] Roger B, Moisand A, Amalric F, Bouvet P (2003). Nucleolin provides a link between RNA polymerase I transcription and pre-ribosome assembly. Chromosoma.

[53] Ginisty H, Sicard H, Roger B, Bouvet P (1999). Structure and functions of nucleolin. Journal of cell science.

[54] Watanabe-Susaki K (2014). Biosynthesis of ribosomal RNA in nucleoli regulates pluripotency and differentiation ability of pluripotent stem cells. Stem Cells.

[55] Barneche F, Steinmetz F, Echeverria M (2000). Fibrillarin genes encode both a conserved nucleolar protein and a novel small nucleolar RNA involved in ribosomal RNA methylation in Arabidopsis thaliana. The Journal of biological chemistry.

[56] Schimmang T, Tollervey D, Kern H, Frank R, Hurt EC (1989). A yeast nucleolar protein related to mammalian fibrillarin is associated with small nucleolar RNA and is essential for viability. EMBO J.

[57] Smetana, K. & Busch, H. The nucleolus and nucleolar DNA. *In: Busch H (ed) The cell nucleolus*. *Academic*, *New York* **1**, 74 (1974).

[58] Shaw P, Doonan JH (2005). The Nucleolus. Playing by different rules?. Cell Cycle.

[59] Stepinski D (2014). Functional ultrastructure of the plant nucleolus. Protoplasma.

[60] Zhu J (2008). Involvement of Arabidopsis HOS15 in histone deacetylation and cold tolerance. Proc Natl Acad Sci USA.

[61] Agius F, Kapoor A, Zhu JK (2006). Role of the Arabidopsis DNA glycosylase/lyase ROS1 in active DNA demethylation. Proc Natl Acad Sci USA.

[62] Sridha S, Wu K (2006). Identification of AtHD2C as a novel regulator of abscisic acid responses in Arabidopsis. The Plant journal: for cell and molecular biology.

[63] Luo M (2012). HD2C interacts with HDA6 and is involved in ABA and salt stress response in Arabidopsis. J Exp Bot.

[64] Sieberer BJK, Franssen-Verheijen H, Emons T, Vos AM, Cell JW (2009). proliferation, cell shape, and microtubule and cellulose microfibril organization of tobacco BY-2 cells are not altered by exposure to near weightlessness in space. Planta.

[65] Sieberer B, Emons A, Vos J (2007). culturing immobilized plant cells for the TUBUL space experiments on the DELTA and 12S Mission. Microgravity Sci Technol.

[66] Kamal KY, van Loon JJWA, Medina FJ, Herranz R (2017). Embedding Arabidopsis plant cell suspensions in low-melting agarose facilitates altered gravity studies. Microgravity Science and Technology.

[67] Herranz R, Valbuena MA, Manzano A, Kamal KY, Medina FJ (2015). Use of microgravity simulators for plant biological studies. Methods Mol Biol.

[68] Manzano A (2018). Novel, Moon and Mars, partial gravity simulation paradigms and their effects on the balance between cell growth and cell proliferation during early plant development. npj Microgravity.

[69] Gonzalez-Camacho F, Medina FJ (2006). The nucleolar structure and the activity of NopA100, a nucleolin-like protein, during the cell cycle in proliferating plant cells. Histochemistry and cell biology.

[70] Testillano PS, Gonzalez-Melendi P, Ahmadian P, Risueño MC (1995). The methylation-acetylation method: an ultrastructural cytochemistry for nucleic acids compatible with immunogold studies. Journal of structural biology.

[71] Steel, R. G. D. & Torrie, J. H. *Principles and Procedures of Statistics*. *A Biometrical Approach*. 2nd Ed. edn, (MacGraw Hill Book Company, 1980).

