# Supplementary material for: Simulated microgravity, Mars gravity, and 2g hypergravity affect cell cycle regulation, ribosome biogenesis, and epigenetics in Arabidopsis cell cultures
Source: Sci Rep. 2018 Apr 23;8:6424. doi: 10.1038/s41598-018-24942-7 (PMC5913308; doi:10.1038/s41598-018-24942-7)
Supplement: Supplementary file 1 — Supplementary information [file 41598_2018_24942_MOESM1_ESM.docx]

**Simulated microgravity, Mars gravity, and 2g hypergravity affect cell cycle regulation, ribosome biogenesis, and epigenetics in Arabidopsis cell cultures**

^1,2^Khaled Y. Kamal*, ^2^Raúl Herranz, ^3,4^Jack J.W.A. van Loon, ^2^F. Javier Medina

^1^Agronomy department, Faculty of Agriculture, Zagazig University, Zagazig, Egypt

^2^Centro de Investigaciones Biológicas (CSIC), Ramiro de Maeztu 9, 28040 Madrid, Spain

^3^DESC (Dutch Experiment Support Center), Dept. Oral and Maxillofacial Surgery / Oral Pathology, VU University Medical Center & Academic Centre for Dentistry Amsterdam (ACTA), Gustav Mahlerlaan 3004, 1081 LA Amsterdam, The Netherlands.

^4^ESA-ESTEC, TEC-MMG, Keplerlaan 1, NL-2200 AG, Noordwijk, The Netherlands


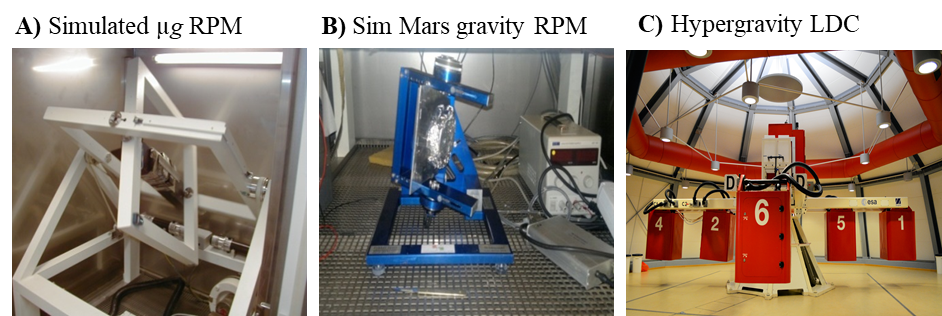


**Supplementary Figure 1. Experiments on the Random Positioning Machine (RPM) and the Large Diameter Centrifuge (LDC) at ESA-ESTEC.** The RPM can be used for both simulated microgravity (Sim µ*g*) and simulated Mars gravity (Mars gravity, 0.37*g*) while the LDC is used for generating hypergravity (e.g., 2*g*). **A)** The Random Position Machine (RPM) with two independently driven perpendicular frames located inside an incubator based at the LIS Laboratory of ESTEC-ESA. **B)** Desktop RPM is suitable to be used within a standard incubator. This desktop RPM is used to simulate microgravity and partial gravity using specific software (RPM^SW^) controlling the speed, axis angles (see, (Manzano et al., Submitted). Experimental samples are placed in the center between the two axes to increase the quality of the simulated microgravity (Hasenstein and van Loon, 2015; van Loon, 2007). **C)** Large Diameter Centrifuge (LDC) has been developed by ESA as a complement to the micro-/partial gravity RPM facilities. For this study, we applied hypergravity of 2*g*. It has four arms, each of which can support two gondolas. The rotation of the LDC was set such that it creates the exact hypergravity field at the location of experiment site inside each gondola (Van Loon et al., 2008).


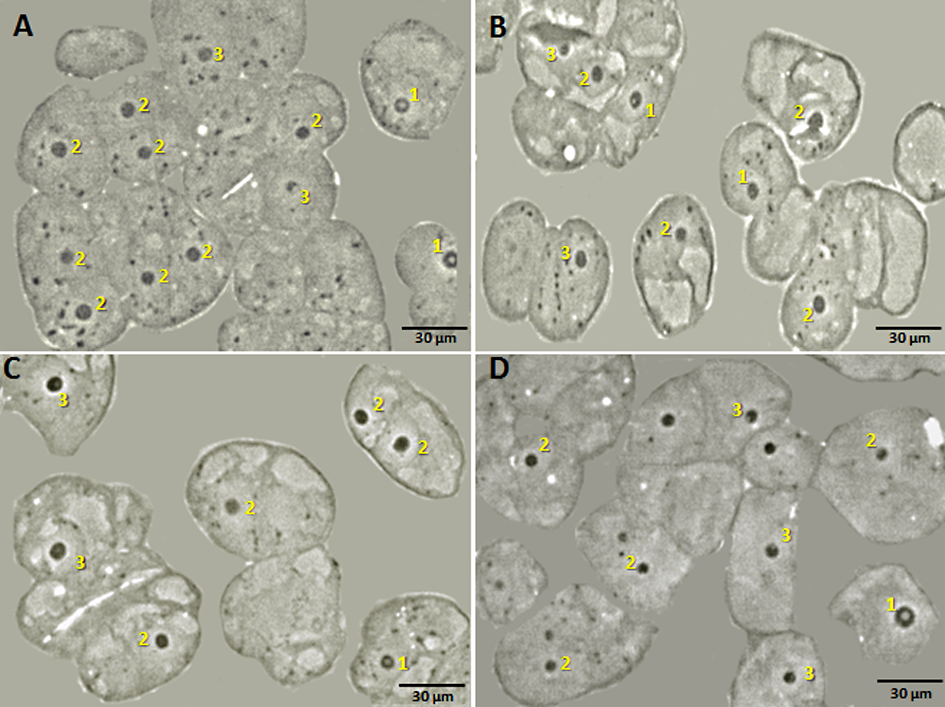


**Supplementary Figure 2: Nucleolar structural models observed in phase-contrast microscopical images of cultured cells after 3 hours exposure to different conditions of gravity.** A) 1*g* control; B) Simulated µ*g*; C) Simulated Mars gravity, D) 2g hypergravity. The different nucleolar models, as defined by Manzano (Manzano et al., 2016) are identified and labeled by numbers: (1) Vacuolated, (2) Compact, (3) Fibrillar.


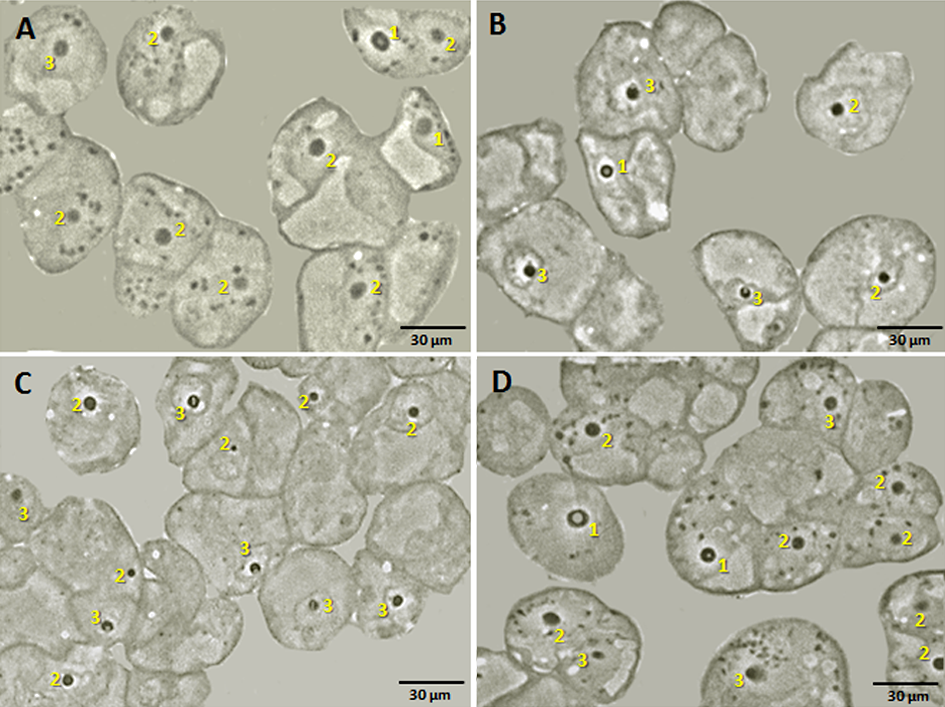


**Supplementary Figure 3: Nucleolar structural models observed in phase-contrast microscopical images of cultured cells after 14 hours exposure to different conditions of gravity.** A) 1g control; B) Simulated µg; C) Simulated Mars gravity, D) 2g hypergravity. The different nucleolar models, as defined by Manzano (Manzano et al., 2016) are identified and labeled by numbers: (1) Vacuolated, (2) Compact, (3) Fibrillar.


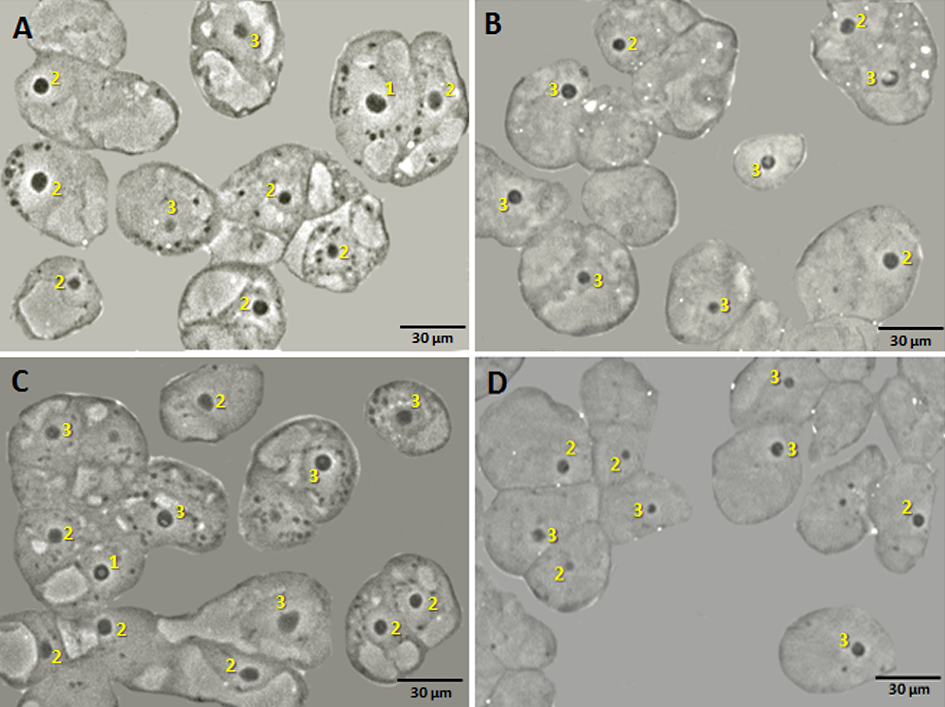


**Supplementary Figure 4: Nucleolar structural models observed in phase-contrast microscopical images of cultured cells after 24 hours exposure to different conditions of gravity.** A) 1*g* control; B) Simulated µ*g*; C) Simulated Mars gravity, D) 2g hypergravity. The different nucleolar models, as defined by Manzano (Manzano et al., 2016) are identified and labeled by numbers: (1) Vacuolated, (2) Compact, (3) Fibrillar.


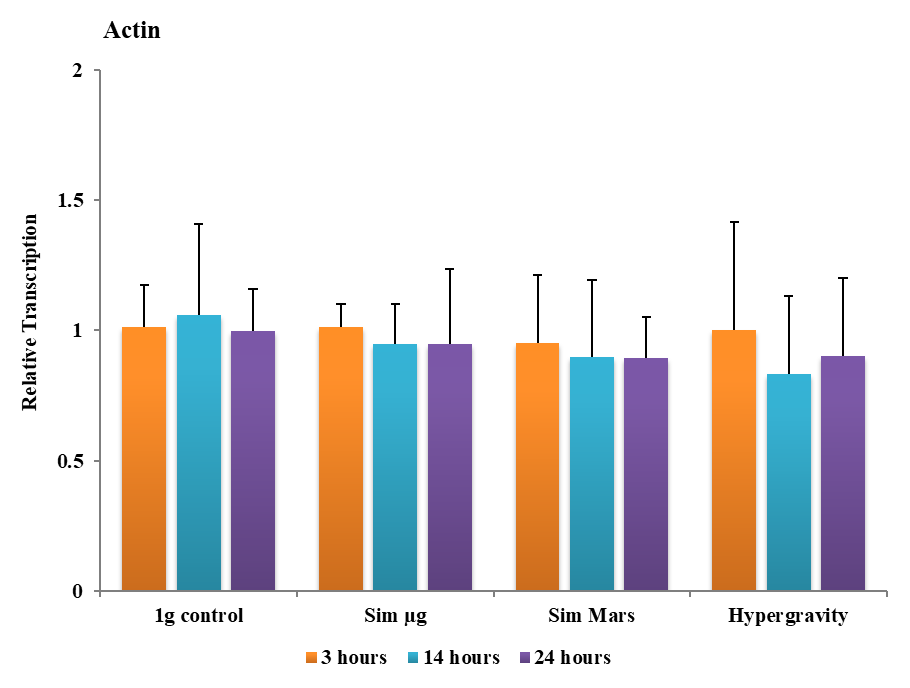


**Supplementary Figure 5. Changes in the Actin reference gene transcription induced by altered gravity levels, estimated by qPCR.** The selected gene (Actin), taken as a reference gene for the qPCR analysis. Cells were exposed to different altered gravity levels: simulated microgravity (Sim µ*g)*, simulated Mars gravity, and 2g hypergravity, for three exposure durations: 3, 14, and 24 hours, and compared to the 1*g* control. Data shown mean ± SE of the three replicates. There were no significant differences between the groups.

| Supplementary table 1: List of the specific antibodies used in the immunofluorescences bases-analysis with the references and its dilutions in the blocking solutions.  *Note:* Dilution percentage is equal in all the antibodies (1:1000) in primary and (1:100) in secondary antibodies except 5mdc (1:25) for DNA methylation and AcH4 (1:50) for histone acetylation. Thus the number of washing should be increased to 5 times more in these two antibodies compared with 3 times in the rest of the antibodies. | | |
| --- | --- | --- |
| Protein of interest | **Specific antibodies** | |
|  | **Primary (1:1000)** | **Secondary (1:100)** |
| Nucleolin L1 | Rabbit polyclonal Ani-AtNUC-L1 (1:1000)  *Provided by Dr. Julio Sáez Vásquez* | Anti-Rabbit  (Alexa 488-Green) |
| Fibrillarin | Mouse monoclonal Anti-Fibrillarin (1:1000)  *Abcam, ab4566, Cambridge, UK* | Anti-Mouse  (Alexa 488-Green) |
| Cyclin B1 | Goat polyclonal anti B-like cyclin (1:1000)  *Santa cruz, sc-12859, Texas, USA* | Anti-Goat  (Alexa 488-Green) |
| Prolifera | Goat polyclonal anti Prolifera (1:1000)  *Santa cruz, sc-12853, Texas, USA* | Anti-Goat  (Alexa 488-Green) |
| DNA methylation | Mouse Monoclonal Anti 5-Methylcytidine (1:25)  *Eurogentec , BI-MECY-0500, Belgium* | Anti-Mouse  (Alexa 488-Green) |
| Histone Acetylation | Rabbit Polyclonal Anti acetyl-Histone H4 (1:50)  *Milipore, Cat.# 06-866, Temecula, USA* | Anti-Mouse  (Alexa 488-Green) |

| Supplementary table 2: Sequence of the different primers (5´-3´) used to follow the expression of key functions genes | | | |
| --- | --- | --- | --- |
| Functions | **Interested Genes** | **Primer (Forward)** | **Primer (Reverse)** |
| Ribosome biogenesis | ***AtNUC-L1*** (At1g48920) | ATGGGAAAGTCTAAATCCGC | TCCACGACC ACGATCACT T |
| Cell cycle | ***Prolifera PRL*** (At4g02060) | TGGGTGGAAGAGGAAAATTG | CTGGCTCCTTCATCCTTCAG |
| Epigenetics | ***MET1*** (At5g49160) | GCTTAATCCAGCCCAGCATA | CACCTTTACCAGCAGCCTTC |
| Reference | ***Actin***  (At3g18780) | GCACCCTGTTCTTCTTACCG | ATCCAGCACAATACCGGTTGTA |

| **Supplementary table 3. Distribution of cell cycle phases under simulated microgravity (Sim µ*g*), simulated Mars gravity, and 2g hypergravity, compared to 1*g* control,** for different times of exposure of the cell culture to each gravitational condition: A) 3 hour. B) 14 hour. C) 24 hour. The relative percentages of the three cell cycle phases were obtained from graphics of DNA content after flow cytometry analysis of cells labeled with DAPI. The proportion of cells in G1 and G2/M phases were quantified from the respective G1 and G2/M peaks of the flow cytometry graphic diagrams, whereas the S-phase percentage was estimated as the remaining proportion of cells up to 100%. 10000 cells in three replicates were counted by flow cytometry. Significant differences versus 1g control are shown (*). P-value > 0.05. SE values for the experimental error is estimated using three different biological replicates. | | | | |
| --- | --- | --- | --- | --- |
|  | | **Relative percentages of the three cell cycle phases** | | |
|  |  | **G1 Phase**  **(Mean ±SE)** | **S Phase**  **(Mean ±SE)** | **G2/M Phase**  **(Mean ±SE)** |
| A) 3 hours experiment | **1g control** | **64.6 ± 0.69** | **12 ± 1.00** | **23.4 ± 1.63** |
|  | Sim µg | 69 ± 4.00 | 12.4 ± 2.26 | 18.6 ± 6.26 |
|  | Sim Mars | 61.6 ± 2.25 | *19.6 ± 3.33 | 18.8 ± 4.72 |
|  | Hypergravity | 64 ± 1.00 | 15.2 ± 4.01 | 20.8 ± 3.27 |
| B) 14 hours experiment | **1g control** | **67.8 ± 3.42** | **10.2 ± 3.67** | **22 ± 2.61** |
|  | Sim µg | 52.8 ± 5.31 | *23.6 ± 1.91 | 23.6 ± 4.71 |
|  | Sim Mars | 67 ± 2.65 | *17.2 ± 2.76 | 15.8 ± 1.15 |
|  | Hypergravity | 55.6 ± 1.81 | *20.4 ± 2.43 | 24 ± 1.75 |
| C) 24 hours experiment | **1g control** | **62 ± 1.73** | **17.9 ± 1.61** | **20.1 ± 2.55** |
|  | Sim µg | *50.4 ± 2.96 | *28.2 ± 3.00 | 21.4 ± 0.17 |
|  | Sim Mars | *53.8 ± 3.67 | 18.4 ± 2.61 | *27.8 ± 5.88 |
|  | Hypergravity | 66.8 ± 1.71 | 14 ± 1.37 | 19.2 ± 3.05 |

**References**

Hasenstein, K. H., and van Loon, J. J. W. A. (2015). Clinostats and other rotating systems — Design, function, and limitations. in “Generation and Applications of Extra-Terrestrial Environments on Earth”, D.A. Beysens & J.J.W. A. van Loon (eds.). . *River Publishers, Denmark.*

Manzano, A., Herranz, R., den Toom, L. A., te Slaa, S., Borst, G., Visser, M., Medina, F. J., and Loon, J. v. (Submitted). Novel, Moon and Mars, partial gravity simulation paradigms and their effects on the balance between cell growth and cell proliferation during early plant development. *NPJ microgravity*.

Manzano, A. I., Herranz, R., Manzano, A., Van Loon, J. J. W. A., and Medina, F. J. (2016). Early effects of altered gravity environments on plant cell growth and cell proliferation: Characterization of morphofunctional nucleolar types in an Arabidopsis cell culture system. *Frontiers in Astronomy and Space Sciences* **3**.

van Loon, J. J. W. A. (2007). Some history and use of the Random Positioning Machine, RPM, in gravity related research. *Adv Space Res* **39**, 1161-5.

Van Loon, J. J. W. A., krause, J., Cunga, H., Goncalves, J., Almeida, H., and Schiller, P. (2008). The Large Diameter Centrifuge, LDC, for life and physical sciences and technology. *Proc. of the 'Life in Space for Life on Earth Symposium', Angers, France, 22–27 June 2008. ESA SP-663, December 2008.*
